# Controlling the Friction of Gels by Regulating Interfacial Oxygen During Polymerization

**DOI:** 10.1007/s11249-021-01459-1

**Published:** 2021-06-02

**Authors:** Rok Simič, Nicholas D. Spencer

**Affiliations:** Laboratory for Surface Science and Technology, Department of Materials, ETH Zürich, Zurich, Switzerland

**Keywords:** Hydrogels, Friction, Tribology, Surfaces, Free-radical polymerization, Oxygen

## Abstract

Hydrogel surfaces are of great interest in applications ranging from cell scaffolds and transdermal drug-delivery patches to catheter coatings and contact lenses. In this work, we propose a method to control the surface structure of hydrogels, thereby tailoring their frictional properties. The method is based on oxygen inhibition of the free-radical polymerization reaction during synthesis and enables (i) control of friction over more than an order in magnitude and (ii) spatial control of friction as either a continuous gradient or a distinct pattern. The presented method has successfully been applied to acrylamide-, diacrylate- and methacrylate-based gels, illustrating the universality of the presented method, and its potential use in the above-mentioned applications.

**Graphical Abstract:**

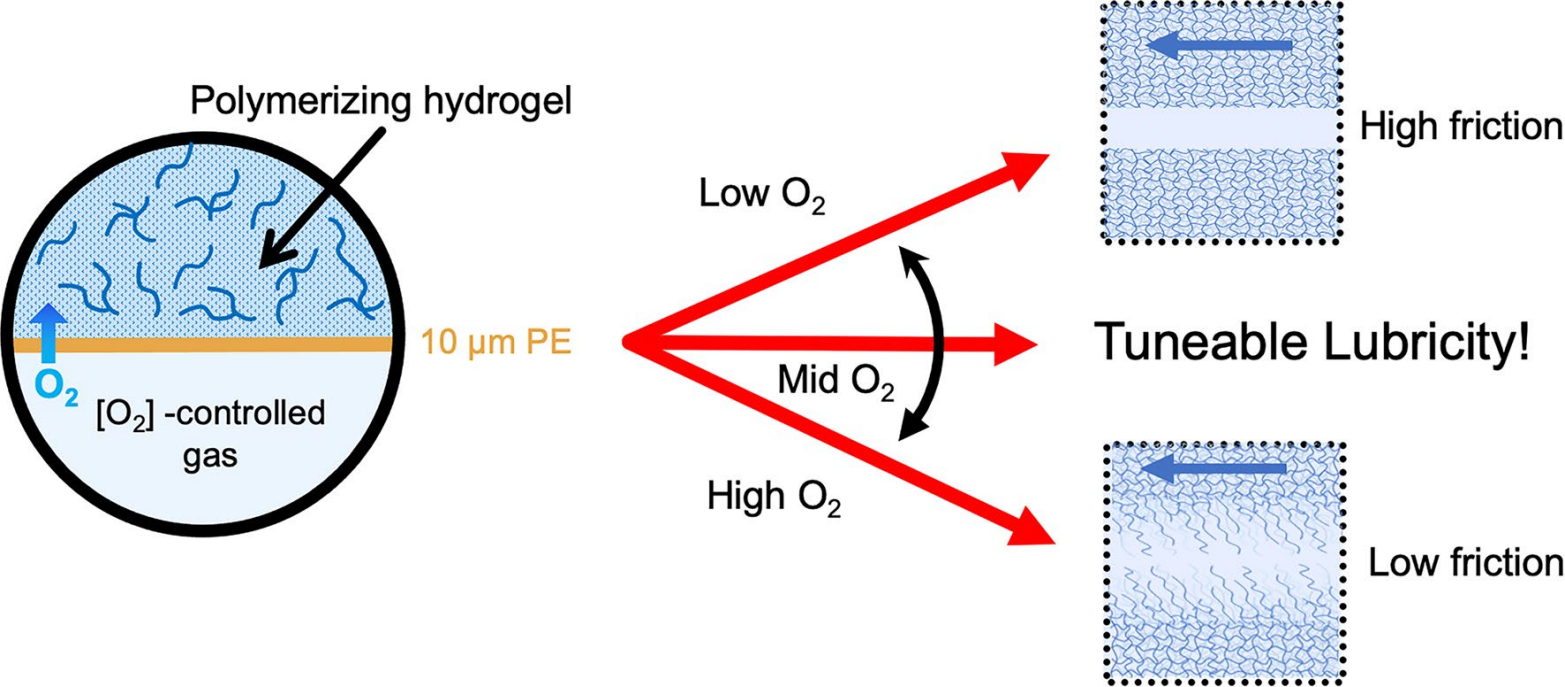

## Highlights

- Oxygen can be used to control structural and therefore tribological properties of hydrogel surfaces.
- A method for controlling hydrogel surface structure and friction is presented.
- The method enables the spatial control of hydrogel surface structure and friction.
- The method is applicable to a wide range of hydrogel types.

## Introduction

Hydrogels, with their cross-linked polymer network and large water content, share many structural similarities with biological tissues. For this reason, hydrogels have often been applied in tissue engineering [1–3], in regenerative medicine [4] and as adhesive, transdermal drug- delivery patches [5]. The high water concentration not only enables high drug and oxygen transmissivity, but can also lead to significant lubricity—important to applications involving sliding contacts, such as catheter coatings and soft contact lenses [6, 7].

The lubricity of hydrogels is usually enhanced by increasing the amount of water at the surface, which can be performed via various physical or chemical methods [8–12]. While most of these approaches involve additional surface treatments, such as growing hydrophilic polymer chains from the surface of an already-synthesized gel, some methods enable the fabrication of gradients in water content, moving outwards to the hydrogel surfaces, within a single synthesis step. The outer edge of such gradients is highly enriched in water and thus exceptionally slippery. This phenomenon, first reported by the group of Gong when synthesizing hydrogels against polymeric molds [8], has been termed “the mold effect” or “the hydrophobic effect” due to the hydrophobic nature of the polymeric molds used. We have recently shown that the observed effect and thus the surface structure and the friction do not completely correlate with mold hydrophobicity [9]. Using a systematic series of experiments, we were able to demonstrate that the effect is due to molecular oxygen, which can diffuse out of many polymeric materials during synthesis, inhibiting polymerization, and especially cross-linking, near the hydrogel-mold interface [13]. The oxygen inhibition of radical polymerization at the interface thus results in incomplete network formation, creating a sparse, water-rich surface region with a high concentration of dangling chains. Such surfaces have been shown to exhibit up to an order of magnitude lower friction compared to hydrogels with a uniform level of cross-linking from the bulk to the surface [14, 15].

Oxygen inhibition of free-radical polymerization is not limited to hydrogels, but is known in multiple areas, including sealants [16], adhesives [17], coatings [18], microfluidics and photolithography [19]. Oxygen-inhibited, incompletely polymerized surfaces usually suffer from decreased bonding strength and poor mechanical properties, and to avoid such detrimental effects, several physical and chemical approaches for preventing oxygen inhibition have been described [20]. Although the phenomenon is detrimental in certain cases, it appears to be beneficial in others, such as for continuous 3D printing [21] or high-throughput microparticle lithography [19]. While oxygen inhibition is well known and has been widely studied, there are practically no examples of its controlled usage to tailor the surface properties of polymers, especially hydrogels.

In this work, we present a method that enables the amount of oxygen delivered to the mold-solution interface to be controlled during radical polymerization, allowing hydrogel friction to be fine-tuned within a range that spans more than one order of magnitude. In the first part we explain the method of controlling the amount of friction, while in the second part we show an example where friction can be spatially controlled. We have used three different types of gel-forming monomers, in order to demonstrate the universality of the approach.

## The Method

**Fig. 1:**
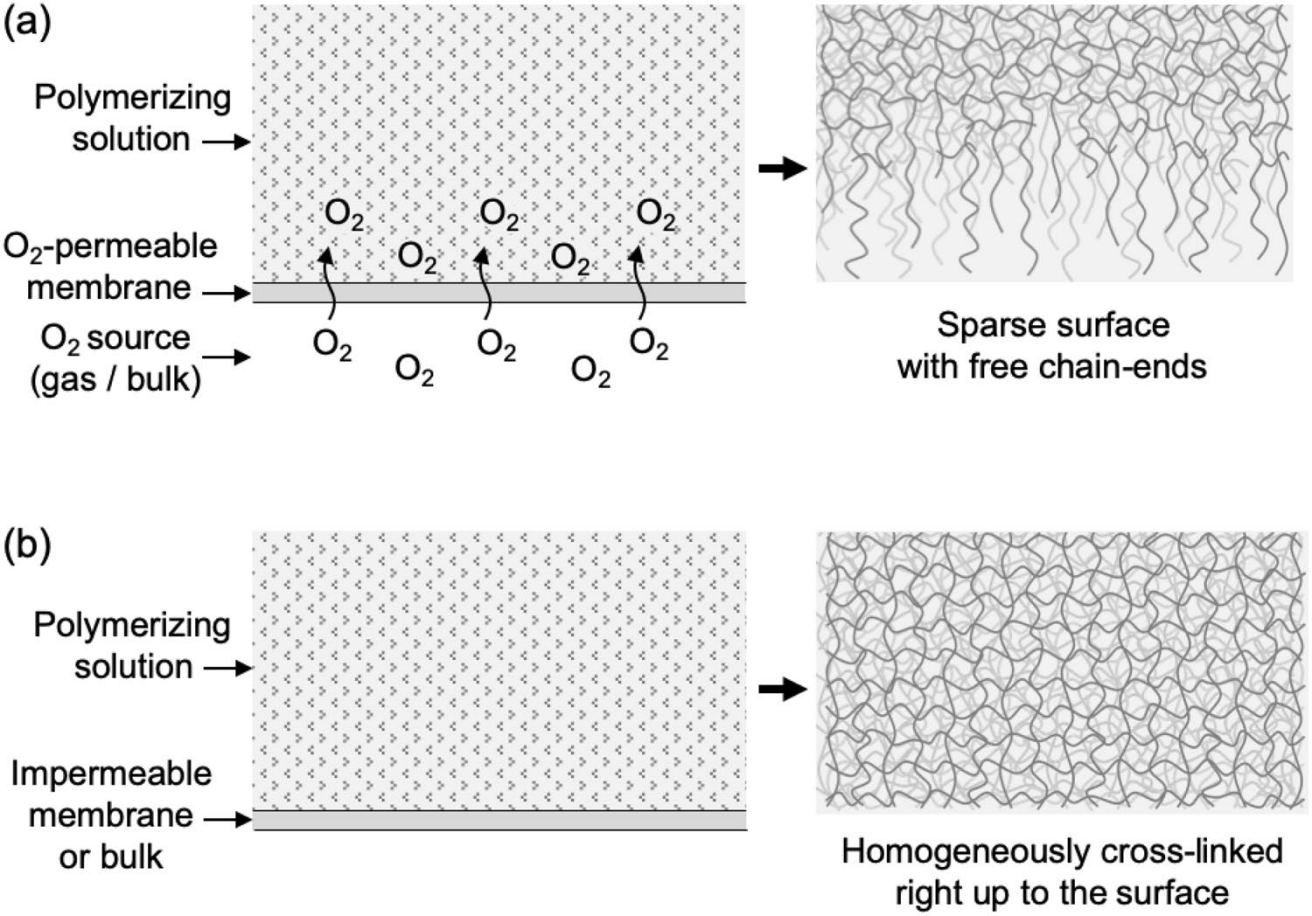
The principle of creating slippery hydrogel surfaces by oxygen inhibition. **a** Molecular oxygen can diffuse into the polymerizing solution through an O_2_-permeable membrane from an oxygen-containing gas or directly out of a bulk mold consisting of an oxygen-permeable material. The increased amount of oxygen at the interface results in a hydrogel with a sparse surface structure. Controlling the amount of molecular oxygen at the interface allows the degree of cross-linking near the surface to be tuned. **b** Polymerizing the solution in the absence of O_2_, e.g. against an O_2_-impermeable membrane or bulk material, such as glass, results in a hydrogel with a homogeneously cross-linked structure

The method is based on our recently published principle of oxygen inhibition of hydrogel radical polymerization near a molding surface [13]. Although the polymerizing solution usually contains a small amount of oxygen, this becomes evenly consumed at an early stage of the reaction without affecting the homogeneity of the network structure. On the other hand, a substantially increased amount of interfacial oxygen near the mold results in the formation of a sparse hydrogel surface. While oxygen is known to react with active radicals, cross-linking is more susceptible to oxygen than chain propagation, since to be effective, two radicals need to be formed on the cross-linking species. Therefore, hydrogel surfaces formed in the presence of oxygen have a low level of cross-linking and thus consist of dangling chain ends, Fig. 1a.

Oxygen can be supplied to the interfacial area from a source gas mixture through an oxygen-permeable membrane, Fig. 1a. Alternatively, oxygen-permeable bulk materials, such as most polymers, contain large amounts of molecular oxygen when equilibrated with ambient air and can also be used as molding materials. The oxygen that diffuses through the membrane or out of the mold during gelation thus inhibits the radical polymerization reaction at the interface, resulting in sparse, soft hydrogel surfaces. The amount of oxygen reaching the reaction mixture through a membrane can be controlled by the composition of the source gas on the other side of the membrane. In the case of bulk molds, the type and the thickness of the permeable material (covering an impermeable substrate such as glass) determines the available amount of molecular oxygen.

In order to produce hydrogels with homogenous structural profiles and cross-linked surfaces, no oxygen should be supplied to the interface during polymerization, Fig. 1b. This can be achieved using an oxygen-free source gas, by deoxygenating bulk polymeric molds, by using oxygen-impermeable mold coatings [e.g. polyvinylidene-chloride (PVDC)] or simply using oxygen-impermeable bulk molds (e.g. glass).

### Controlling the Friction Value

**Fig. 2:**
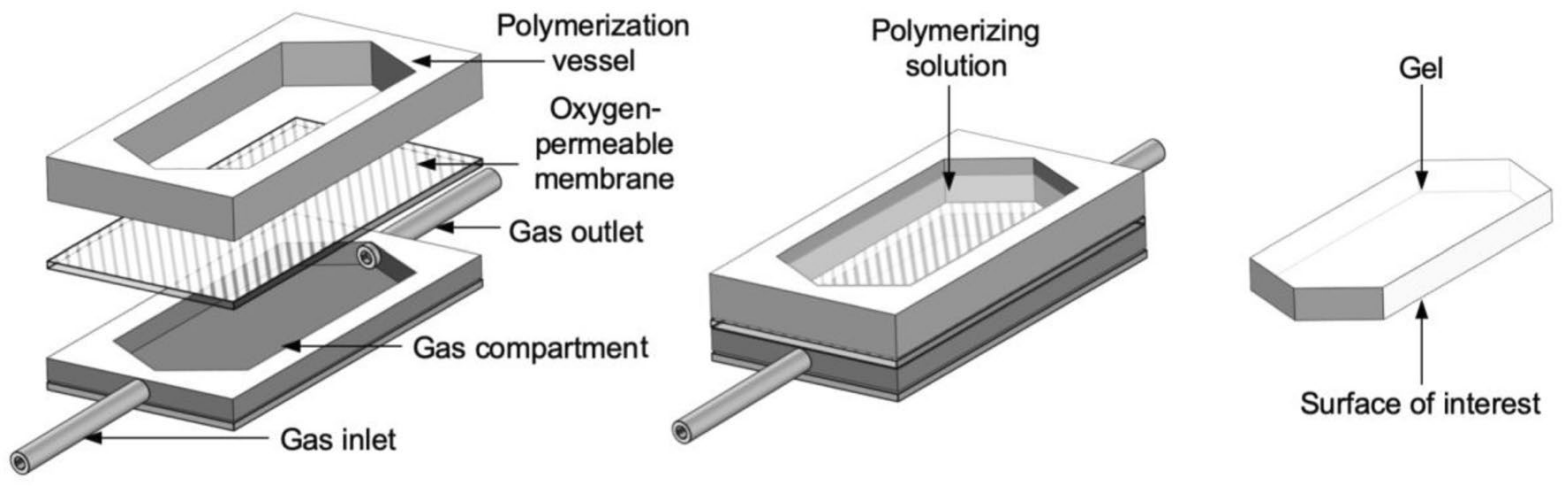
Schematics of the device for synthesizing hydrogels against an oxygen-permeable membrane. Left image shows the exploded view of the device, the middle image shows the assembled device with the polymerizing solution and the image on the right shows an example of a synthesized hydrogel with the surface of interest underneath

In this part, we present a device that enables the surface structure and thus the friction of hydrogels to be tuned by controlling the amount of molecular oxygen that diffuses through an oxygen-permeable membrane to the polymerizing solution. For this, an 11-µm-thick low-density polyethylene (PE) foil (commercially available domestic food wrap, Tangan N°11, Migros, Switzerland) was used as the membrane to separate the polymerizing solution from mixtures of gaseous oxygen and nitrogen. Although the oxygen permeability for this particular PE foil was not determined, we assumed a permeability of common LDPE foils, which is about 8500 cm^3^·25 μm/(m^2^·24 h·atm) at 23 °C and 0% relative humidity (RH) [22]. The surface roughnesses of such films are on the order of tens of nm and thus considerably less than the surface layer thickness of the produced hydrogels [9]. The PE membrane was held in place between two laser-cut polymethylmethacrylate (PMMA) plates of equal lateral dimensions, Fig. 2. Both PMMA plates had an elongated opening measuring 20 mm in width and 50 mm in length. The top plate formed a side-wall of height 5 mm that served as a reaction vessel. The 2-mm-thick bottom plate was covered underneath and was connected to tubes at both ends to form a gas compartment. The gas flow rate was controlled by a syringe pump and set to 5 ml/min, corresponding to 2 mm/s of mean gas velocity within the gas compartment. The amount of oxygen in the flowing gas was varied from 0 to 22%, with the rest consisting primarily of nitrogen. The different amounts of oxygen in the flowing gas were achieved by mixing the corresponding amounts of pure molecular nitrogen and ambient air (~ 50% RH) and the gas was at room temperature (23 °C). After flowing the gas of desired composition through the gas compartment for 2–3 min, the reaction vessel was filled with four milliliters of polymerizing solution. After introducing the solution, the polymerization was carried out as described in the Hydrogel synthesis section. In the Results section we show the indentation results and the coefficients of friction for three different hydrogel types produced with various flowing gas compositions.

### Spatial Control of Friction

**Fig. 3:**
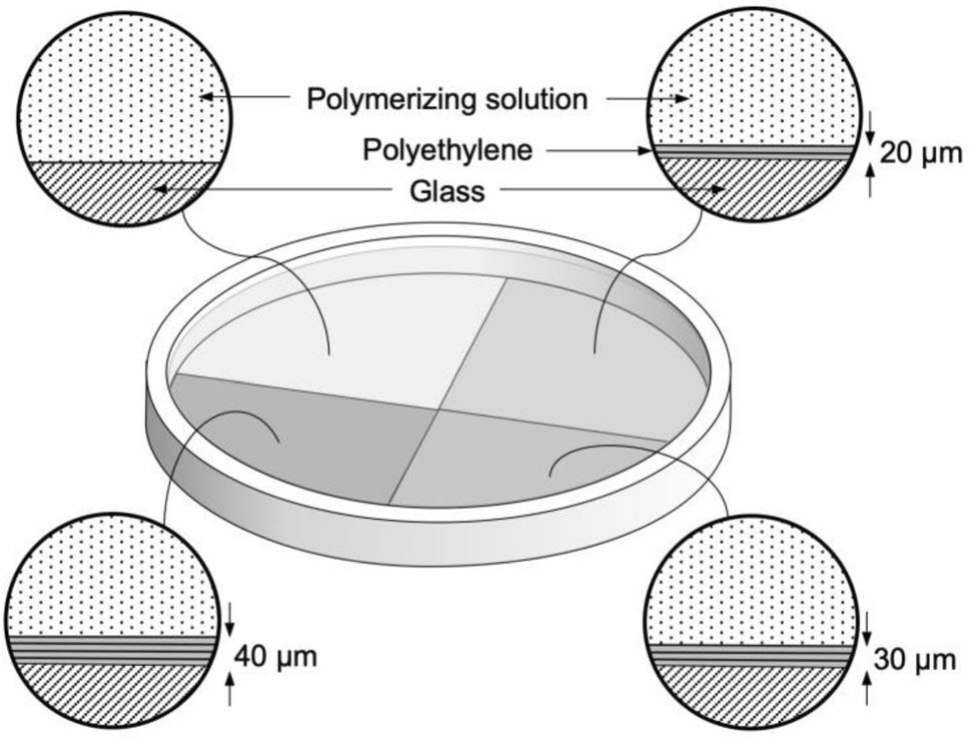
Schematic of a glass petri dish coated with PE layers of different thickness to create hydrogels whose surfaces contain areas with different friction values

In this section, we present an example of a device that enables the production of a hydrogel with distinct regions of different friction. In this case, the mold was a 120-mm-diameter glass petri dish, which is impermeable to oxygen. The inside of the dish was divided into four regions and three of them were covered by 20-, 30- and 40-µm-thick layers of O_2_-permeable, air-equilibrated PE, Fig. 3. Differently to the previous case, where the gas mixture was the source of molecular oxygen, here the air-equilibrated PE layers provide the oxygen to the interface and the supplied amount is determined by the PE layer thickness. To produce similarly thick hydrogel samples as in the previous case, 40 ml of polymerizing solution was poured into the air-equilibrated device and it was polymerized in ambient air as described in the Hydrogel synthesis section.

As a possible alternative to the distinct areas of differently thick, oxygen-saturated layers, a wedge of the same material could be produced to create a hydrogel with continuously changing friction values. Although the gradient, wedge-like layer mold is not presented in this work, it would be a continuous analog to the presented stepped example. Besides PE, polymers such as polypropylene (PP), polyoxymethylene (POM), polystyrene (PS) or Teflon (PTFE) could be used. The approach could also be reversed, and instead of oxygen-saturated polymer coating on top of glass, a thin O_2_-impermeable coating (e.g. PVDC) could be used to block oxygen diffusion from parts of O_2_-saturated polymeric molds [13].

## Experimental

### Hydrogel Synthesis

The polymerizing solution for synthesizing polyacrylamide (PAAm) hydrogels consisted of 9.6 wt.% of acrylamide (> 99%), 0.4 wt.% of *N*,*N*′-methylene-bis-acrylamide (> 99.5%) and 0.01 wt.% of lithium phenyl-2,4,6-trimethylbenzoylphosphinate (LAP) photo-initiator in ultrapure (Milli-Q) water. The LAP photo-initiator was synthesized as described in our previous work [10]. The poly(ethylene glycol) (PEG) hydrogels were synthesized from 10 wt.% of polyethylene-diacrylate (PEGDA, Mn = 700 Da) and 0.01 wt.% of LAP photo-initiator in MilliQ water. Poly(hydroxyethyl methacrylate) (PHEMA) hydrogels were synthesized from 45 wt.% hydroxyethyl methacrylate (HEMA), 15 wt% n-vinyl pyrrolidone (n-VP), 1.2 wt% ethylene glycol dimethacrylate (EGDMA) and 0.1 wt.% of LAP photo-initiator. All components besides the LAP were obtained from Sigma-Aldrich, St. Louis MO, USA.

To prepare the polymerizing solution for the PAAm gels, the monomer and the crosslinker were mixed with the corresponding amount of milli-Q water. Similarly for PEG gels, PEGDA was first mixed with the corresponding amount of milli-Q water. Both mixtures were then sonicated for 5–10 min to completely dissolve the solids. Afterwards, the initiator was added in the respective amount and the solutions were stirred to dissolve the initiator. The solutions were poured in the molds immediately afterwards. No bubbling with N_2_ was used for these two hydrogel types, the dissolved oxygen uniformly present in the solution presumably being rapidly consumed at the beginning of the polymerization reaction. The solutions for the PHEMA gels were prepared by strictly following a recipe from the literature [23]. In this case the mixture of both monomers and the crosslinker was bubbled with N_2_ for 10 min. After adding the initiator, the mixture was bubbled for another 5 min before being poured into the molds.

A UV light source with an intensity of 1.4 mW/cm^2^ at a wavelength of 365 nm (Stratalinker UV Crosslinker 2400, Stratagene Corp., La Jolla, CA, USA) was used to polymerize the hydrogel solutions. The PAAm and PEG hydrogels were exposed to the UV light for 20 min, and the PHEMA gels exposed for 40 min. The polymerization was carried out in ambient air. In the case of PE layers providing the molecular oxygen, equilibration of the layers in air ensures their saturation with molecular oxygen. In the cases where the flowing gas was supplying the oxygen, the evaluated hydrogel surfaces were produced against the permeable membrane and were thus isolated from environmental oxygen. After UV exposure, the synthesized hydrogels were demolded and soaked in Milli-Q water for at least 48 h. Some of the PHEMA gels were afterwards immersed in ethanol for 2–3 h to allow the swelling of the surface region and demonstrate the presence of dangling chains on the surface. This immersion time was chosen to deliberately not swell the bulk completely, and maintain the original size of the gels.

### Nanoindentation

In order to characterize the surface structure of the hydrogels, nanoindentation experiments were performed using an atomic force microscope (AFM, MFP-3D™, Asylum Research, Santa Barbara, USA). A silica microsphere (Kromasil, Nouryon—Separation Products, Bohus, Sweden) with a radius of *R* = 11.5 µm was glued with the help of a 2-component epoxy adhesive (UHU GmbH, Germany) to the end of a tipless cantilever (NSC-36, Mikromash, Bulgaria). The normal spring constant of the bare cantilever *k*_*0*_ was determined before attaching the microsphere by applying the Sader method [24]. By determining the distances from the base of the cantilever to its tip (*L*_*0*_) and to the microsphere (*L*), the effective spring constant was calculated as *k* = *k*_*0*_(*L*_*0*_/*L*)^3^ = 1.61 N/m [25]. After installing the probe and adjusting the laser path, the system was calibrated by pressing the probe against a silicon wafer in water. The force was calculated as *F* = *k x*, and the indentation depth was thus calculated as *d* = *Z−x*, where *Z* is the vertical piezo displacement and *x* is the calibrated cantilever displacement. The contact with the gel in liquid was determined at the point where the force signal began to deviate more than 2σ from the zero-force line, with σ being the standard deviation of the signal noise (≈ 20–30 pN). The threshold value of 2σ has been found as the optimum between detecting a soft surface and excessive scattering of the aligned curves, and has been successfully used in several of our previous studies [9, 14, 26–28]. The approach and retraction speeds were 1 µm/s, showing only a minor hysteresis due to the speed-dependent effects. The measurements were performed at 25 ± 1 °C. Twelve force curves were obtained over an area of 40 × 40 mm^2^ at three different locations of a sample. Graphs show representative force-indentation curves. For isotropic, linearly elastic materials, the Hertzian model is generally valid for strains with *a*/*R* < 0.1, where *a* and *R* are the contact and the probe radii, respectively. For materials with high Poisson ratio of 0.4–0.5, such as the hydrogels used in this work, it has been shown that the Hertzian model is reliable even at *a*/*R* ~ 0.8, which corresponds to indentation depths of *d* ~ 0.64*R* [29]. Due to the gradient structure of some of the gels in this study, we have limited the evaluated depth to *d* ~ 0.1*R* ~ 1.5 µm. Increasing the evaluation depth beyond this limit resulted in worsening of the fit due to the anisotropy of the layers. At the same time, this value corresponded to the maximum indentation depth achieved for the crosslinked PAAm and PEG gels, meaning that the modulus was evaluated over the same depth for all the indented hydrogels. The presented elastic moduli are the average values with errors being one standard deviation of all the measured force curves.

### Friction Measurements

Friction of the hydrogels was measured using a tribometer (CSM Instruments, Peseux, Switzerland). For a flat-pin-on-flat configuration, the pins with a diameter of 10 mm and the larger flat samples were cut from bigger hydrogel slabs and glued to a metal pin holder and PS petri dishes, respectively, using a thin layer of cyanoacrylate-based superglue (Pattex, Henkel AG & Co. KGaA, Düsseldorf, Germany). The hydrogels for the pins were molded against glass and therefore had crosslinked surfaces, while the bottom gel counterparts were produced in various ways, as described above. While flat-on-flat contact was used due to the simplicity of hydrogel fabrication, a sphere-on-flat contact was used in the case of the hydrogel with areas of different friction to enable easier transitioning from one to another area. Tests with PHEMA gels were also performed in a sphere-on-flat configuration to ensure even contact of the less-compliant hydrogels. As shown recently, the two contact configurations have similar friction at sliding speeds used in this work [15]. For a sphere-on-flat configuration, hemispherical gels with a radius of about 9 mm were made in round-bottom glass test tubes and glued to a metal pin holder in the same way as the flat pins. All friction experiments were performed with contact pairs of the same hydrogel material and with contacts fully immersed in liquid. The normal load was set to 0.5 N, corresponding to a contact pressure of about 6–10 kPa for the PAAm and PEG gels, and 30 kPa for the stiffer PHEMA gels. The flat-pin-on-flat contacts were used in combination with reciprocating sliding at a stroke length of 20 mm. The sliding speeds were varied within the range of 0.1–20 mm/s for both contact configurations. A unidirectional sliding configuration with a radius of 15 mm was only used for the hydrogel with differently molded areas, in order to demonstrate the friction during continuous sliding over these areas.

## Results and Discussion

### Controlling Mechanical Properties and the Friction Value

**Fig. 4:**
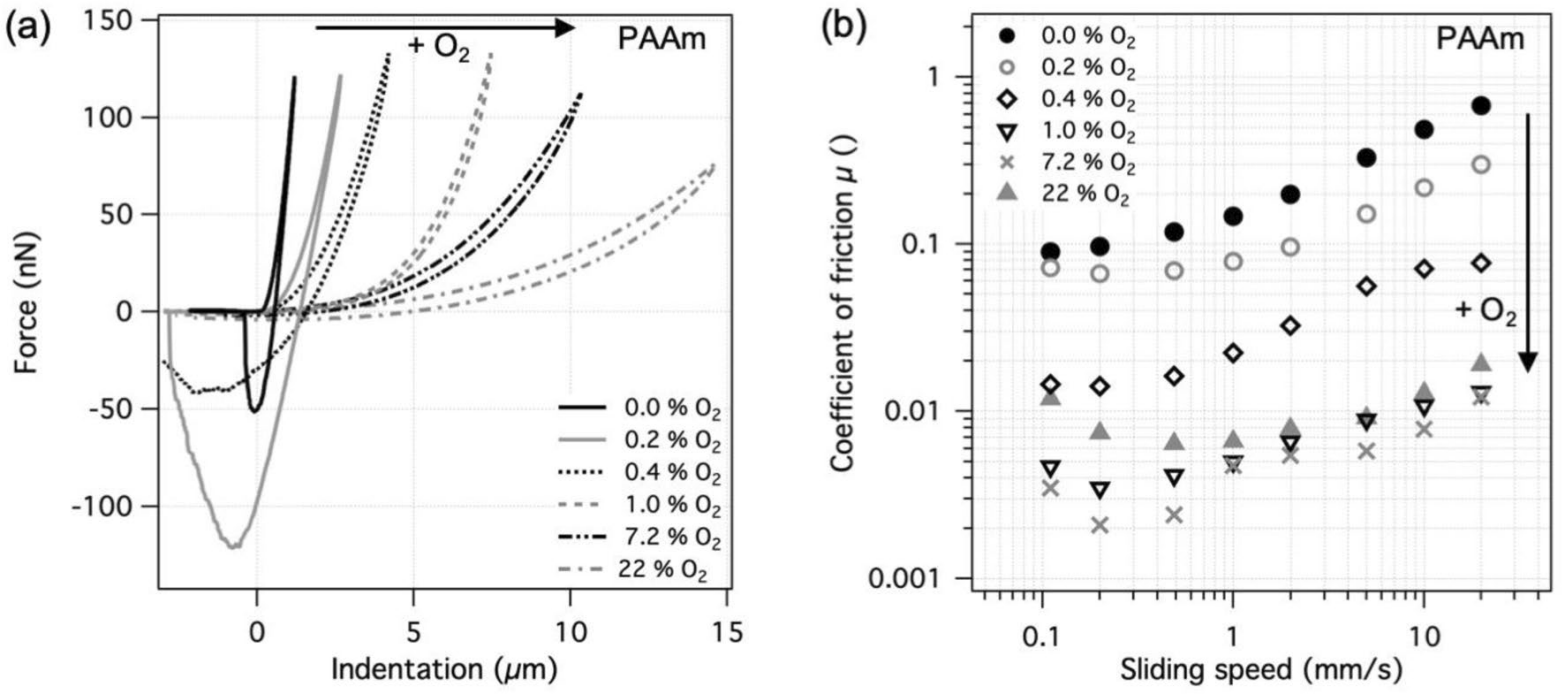
Results for PAAm hydrogel surfaces prepared against a permeable PE membrane, which separates the polymerizing solution from the different gas mixtures. **a** Representative force-indentation curves and **b** coefficient of friction at different sliding speeds in water

The force-indentation curves shown in Fig. 4a were obtained for PAAm hydrogel surfaces synthesized against a thin PE membrane while using different amounts of molecular oxygen in the source gas. The indentation curves obtained on a hydrogel produced with pure nitrogen (0% O_2_) as the source gas showed the stiffest response and could be nicely described by the Hertzian contact model, indicating a homogeneously cross-linked structure throughout the indented depth of the hydrogel. The extracted elastic modulus was 21 ± 1 kPa. Increasing the amount of oxygen in the source gas resulted in progressive softening of the hydrogel surfaces, Fig. 4a. With only 0.2 and 0.4% of O_2_ in the source gas, the elastic modulus at the surface dropped to about 7 and 3 kPa, respectively. At 1% of O_2_, the elastic modulus within the first 1.5 µm was already well below 1 kPa and the estimated thickness of this soft surface layer was more than 5 µm. At 22% O_2_, the surface appeared even softer with thickness of > 15 µm. Such an exceptionally soft response upon indentation is attributed to a sparse hydrogel surface with a very low degree of cross-linking. The polymer density and the degree of crosslinking gradually increase with increasing depth, resulting in a non-Hertzian response during the indentation. Although the thickness of such gradient layers was only roughly estimated from the shape of the indentation curves, the values are very close to recently reported values for similar materials [30].

The observed adhesion on the dense, crosslinked hydrogel surfaces presumably originates from the relatively high contact pressures between the silica probe and the hydrogel surface. Under such conditions the hydrogel structure could partially dehydrate and form hydrogen bonds with the silica. Reduced polymer density and hence lower contact pressure on the sparse hydrogel surfaces could enable better hydration and therefore avoid noticeable adhesion.

Since oxygen permeability through PE increases with temperature in an Arrhenius-like fashion [22, 31], performing the synthesis at higher temperature would allow more oxygen to reach the polymerization solution, which would further reduce the polymerization degree at the hydrogel surface. Increasing humidity of the flowing gas should in theory also increase the permeability of oxygen through PE foil. However, the presence of an aqueous solution on one side of the PE foil is likely to saturate the foil with water molecules, meaning that changing the relative humidity in the flowing gas would have negligible effect on oxygen permeability. In order to prove these assumptions, however, experiments at various temperatures and humidity levels would have to be performed.

Figure 4b shows the coefficient of friction as a function of sliding speed for the same PAAm hydrogels when slid against a flat, cross-linked PAAm pin at a contact pressure of 6 kPa in water. The stiffest hydrogel surface, which was produced in the absence of molecular oxygen, also had the highest coefficient of friction, with values lying between 0.1 and 0.7. Similarly to the elastic modulus at the hydrogel surface, the coefficient of friction decreased with increasing amounts of oxygen in the source, Fig. 4b. At 1% O_2_ and above, which resulted in elastic moduli below 1 kPa, the coefficient of friction was mostly around 0.01 and below. This is over an order of magnitude lower than for the crosslinked hydrogel and is attributed to the larger amount of liquid within the shearing zone during sliding of soft hydrogel surfaces. The adhesion that was observed between the silica probe and the hydrogel during indentation presumably does not occur between two equivalent hydrogel surfaces. Therefore, the frictional dissipation is more likely to come from hydrodynamic shearing and/or polymer fluctuations [14, 32]. These results show that increasing the amount of oxygen in the source gas reduces the modulus of the hydrogel surfaces and decreases their friction.

**Fig. 5:**
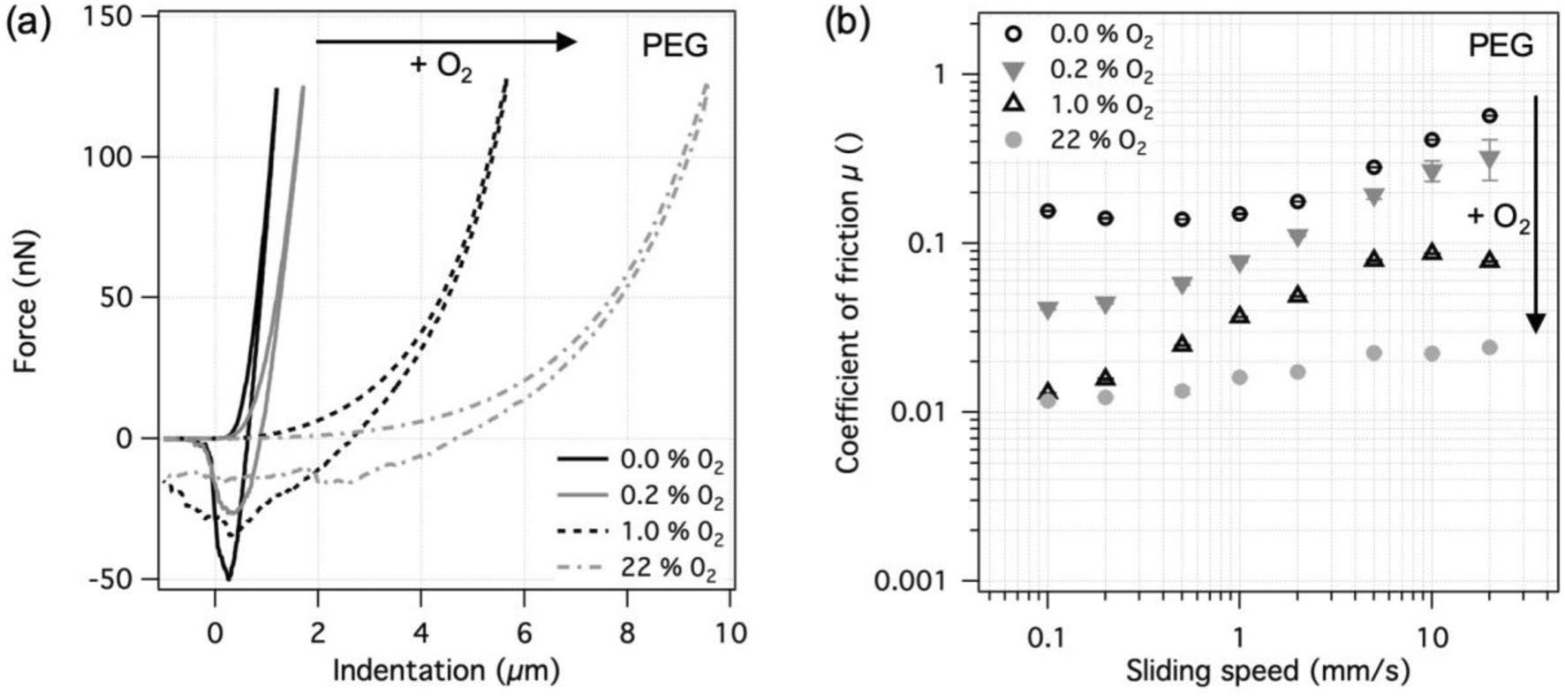
Results for PEG hydrogel surfaces prepared against a permeable PE membrane, separating the polymerizing solution from the different gas mixtures. **a** Representative force-indentation curves and **b** coefficient of friction at different sliding speeds in water

The same experiment as with PAAm hydrogels was performed with PEG hydrogels, Fig. 5. Similarly to PAAm, the PEG hydrogel surface produced in the absence of O_2_ in the source gas appeared to be the stiffest, with an elastic modulus of 26 ± 1 kPa. At 0.2% O_2_ the elastic modulus dropped to 15 ± 1 kPa, indicating a significantly looser surface structure compared to the 0% O_2_ case. Oxygen amounts of 1% and more again resulted in soft surfaces with elastic moduli well below 1 kPa. Analogously, the coefficient of friction decreased by more than an order of magnitude when increasing the amount of oxygen from 0 to 22%, Fig. 5b. The results show that the presented method can be used with similar efficiency for controlling the surface structure and friction of PEG-based hydrogels as well.

**Fig. 6:**
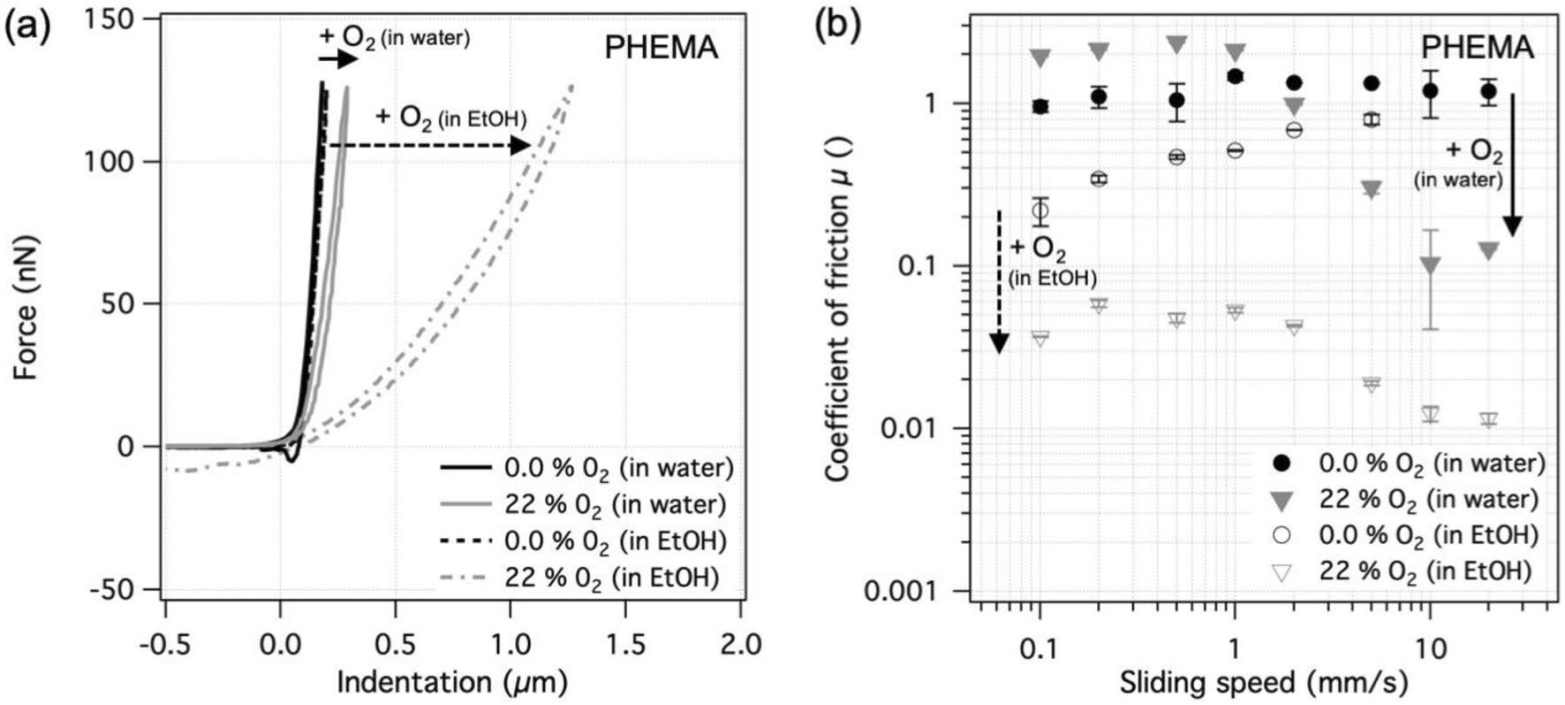
Results for PHEMA gel surfaces prepared against a permeable PE membrane, separating the polymerizing solution from the different gas mixtures. Results for the gels swollen in water and in ethanol are shown. **a** Representative force-indentation curves and **b** coefficient of friction at different sliding speeds in water and in ethanol

Figure 6 shows the indentation and friction results for PHEMA hydrogels synthesized in the reaction vessel with either 0 or 22% of oxygen in the source gas mixture. The elastic modulus at the surface of PHEMA hydrogels synthesized at 0 and at 22% was 480 ± 10 kPa and 190 ± 10 kPa, respectively, which is a relatively small difference considering the effects observed with PAAm and PEG hydrogels. Since PHEMA gels have, in addition to their covalently linked network structure, a secondary structure stabilized by hydrophobic bonding that prevents the surface dangling chains from swelling freely, the observed result is not completely unexpected [33, 34]. Immersing PHEMA gels in ethanol, which replaces the interstitial water, enables additional swelling of the polymer structure due to the better affinity of ethanol towards the hydrophobic moieties of the HEMA polymer [33, 34]. Performing nanoindentation experiments on PHEMA gels in ethanol yielded surface elastic moduli of 440 ± 30 kPa and 13 ± 1 kPa in the case of 0 and 22% oxygen, respectively, Fig. 6a. The small decrease of the modulus and the shape of the indentation curve in the 0% case indicate a homogeneously cross-linked profile, similar to that observed for the PAAm and the PEG hydrogels. In the case of 22% O_2_, however, the drastic decrease in elastic modulus at the surface was caused by substantially better swelling of the oxygen-inhibited, low-cross-linked surface in ethanol compared to water. This result shows that oxygen inhibits the polymerization near the surface for methacrylate-based gels as well, however, in the case of PHEMA the effect is only apparent when solvated with a sufficiently good solvent.

The coefficients of friction for sliding a cross-linked PHEMA hemisphere against the two PHEMA surfaces in water and in ethanol are shown in Fig. 6b. Both types of PHEMA surfaces in water had high coefficients of friction with values mostly between 1 and 2. Such high values are not unexpected, considering the relatively poor solvent quality of water for PHEMA and the rubber-like nature of PHEMA gels [34]. The drop in the coefficient of friction at higher sliding speeds for the oxygen-inhibited gel surface is attributed to the formation of a water-rich shearing zone at the low-cross-linked surface. On the other hand, sliding the same gels solvated in ethanol resulted in a considerable drop in friction for both surface types. The coefficient of friction for the cross-linked surface in ethanol decreased at low speeds when compared to sliding in water, which could be due to the better solvation of the polymer network. The coefficient of friction for the sparse PHEMA surface in ethanol also dropped significantly when compared to sliding in water. Moreover, with the values between 0.01 and 0.05, the coefficient of friction for the sparse PHEMA surface was about an order of magnitude lower compared to the analogous crosslinked gel surface in ethanol. This shows that the presented method can be used not only to affect the surface structure of methacrylate-based gels, but also their friction, especially when adequately solvated.

### Spatial Control of Friction

**Fig. 7:**
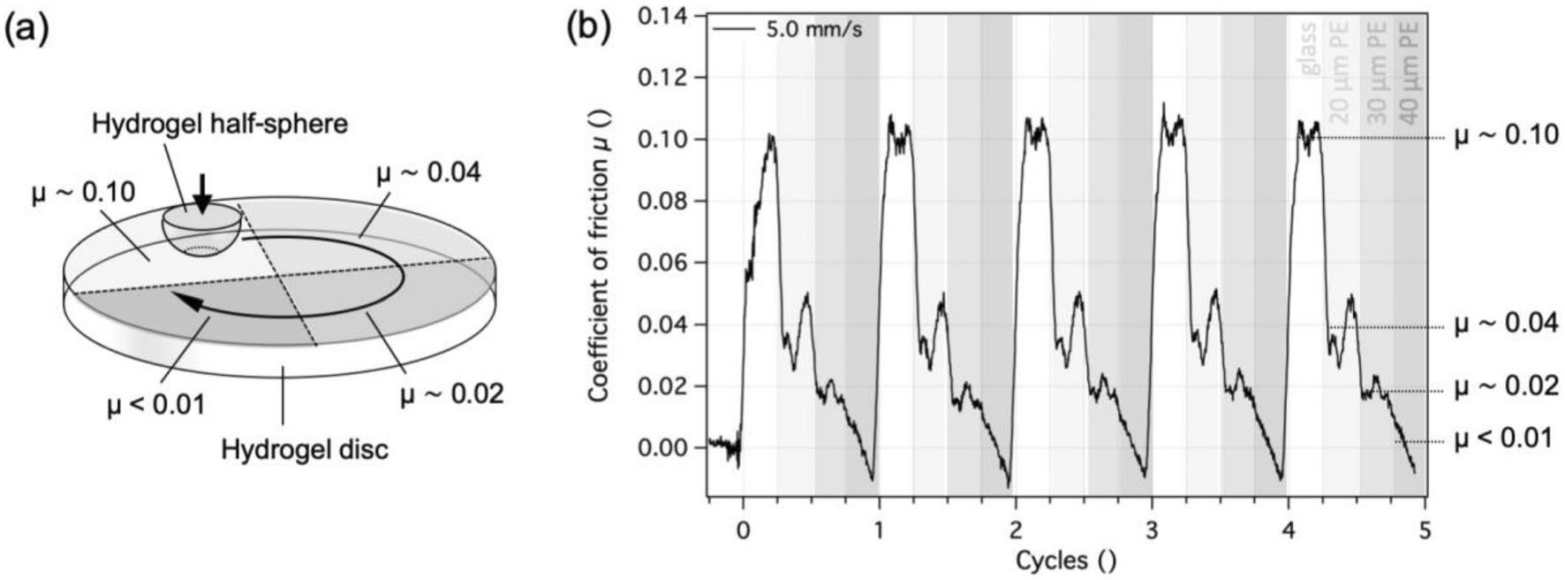
Hydrogel with a spatial variation of friction values. **a** Schematic of unidirectional sliding of a hydrogel half-sphere over a hydrogel disc with four areas of different friction values. The differently shaded segments denote the areas molded against bare glass or against glass with a 20-, 30- or 40-µm-thick PE coating. **b** Coefficient of friction as a function of cycles for a hydrogel sphere sliding successively and repeatedly over the four areas of the PAAm hydrogel disc in water at 5 mm/s

In order to present another way of tailoring friction by oxygen inhibition and to introduce spatial control over the friction values, we have coated distinct areas of a glass petri dish with differently thick PE layers, as shown in Fig. 3. In this case, the air-equilibrated PE layers contain molecular oxygen, and the layer thickness determines the amount of oxygen available for the inhibition of the polymerization reaction near the interface. Therefore, the PAAm hydrogel that was produced in the described mold had four areas with distinctly different surface structures. Unidirectional sliding of a hemispherical PAAm hydrogel over all four areas in one cycle resulted in the expected evolution of the coefficient of friction, as shown in Fig. 7. When the sphere was sliding over the glass-molded PAAm surface at 5 mm/s, the coefficient of friction was about 0.1. Sliding the sphere over the areas molded against the 20-, 30- and 40-µm-thick PE layers, however, resulted in coefficients of friction of about 0.04, 0.02 and less than 0.01, respectively. These values do not take into account the unstable friction values when transitioning between regions. The friction in the slipperiest region appeared lower than the contributions from the topographically uneven surface, which caused the measured friction values to drop below zero. For this reason, the exact value in this region could not be precisely determined and is listed as “ < 0.01”. The results of this experiment show that hydrogels with distinct areas of different friction can be created in one single synthesis step. This approach could be especially suitable for the manufacture of contact lenses for astigmatism, where the varying friction values between an eyelid and the contact lens could help in maintaining the correct orientation of the lens. Moreover, creating a lens with a slippery front side and comparatively higher friction on the reverse side could, for example, help keeping the lens position during a blink of the eye. Since the method enables surfaces to be created with small differences in friction, the friction values on both sides of the lens could be kept low enough to avoid applying excessive shear to the corneal epithelium [35, 36].

## Conclusion

In this work, we have presented a method for controlling hydrogel friction using oxygen inhibition of polymerization during synthesis. Although small amounts of oxygen dissolved in the reaction mixture get consumed during polymerization without affecting the homogeneity of the network, sufficient amounts of oxygen at the mold-solution interface can suppress the polymerization and cross-linking to the point where a soft surface comprised mostly of dangling chains is formed on a hydrogel. Such soft surfaces have low coefficients of friction due to the large amounts of water they provide to the shearing zone in the sliding contact. Controlling the amount of oxygen supplied to the interface of the polymerizing gel thus allows the surface structure and hence the friction to be controlled. In the first part, we have presented a way to control the supply of oxygen by molding a hydrogel against an oxygen-permeable membrane and adjusting the composition of a gas on the other side of the membrane. In the second part, we have used differently thick O_2_-permeable polymer layers on top of glass to define the amount of oxygen available for inhibition. Furthermore, we have shown how this method can be used to gain spatial control over friction. By using three different types of monomers, we have demonstrated that the method is applicable to a wide range of hydrogels, illustrating its applicability in ophthalmology and broader fields of medicine.
